# High‐throughput screening identifies a previously undescribed checkpoint controlling mitotic progression in response to DNA damage

**DOI:** 10.1111/febs.70183

**Published:** 2025-07-10

**Authors:** Nan Li, Rachel Gatenby, Thomas Walne, Caroline Daye, Steven Watson, Priya Lata, Stephen Brown, Ruth Thompson

**Affiliations:** ^1^ Division of Clinical Medicine, Faculty of Health University of Sheffield UK; ^2^ School of Biosciences, Faculty of Science University of Sheffield UK; ^3^ Nucleic Acids Institute University of Sheffield UK

**Keywords:** cell cycle, checkpoint, DNA damage, mitosis, SOD1

## Abstract

Following DNA damage, the cell cycle can be slowed or halted to allow for DNA repair. However, the mechanisms underpinning mitotic delay in response to DNA damage are unclear. Through an unbiased high‐throughput screen, here, we have identified superoxide dismutase 1 (SOD1) as an essential factor mediating mitotic delay in response to DNA damage. Cells with damaged DNA arrest at metaphase, indicating involvement of the spindle assembly checkpoint (SAC); however, this response is lost following SOD1 depletion. Furthermore, whilst depletion of SAC proteins promotes rapid cell division (often less than 10 min) in all conditions, SOD1 depletion has no impact on mitotic progression either in unperturbed mitosis or in response to spindle poisons and does not decrease the mitotic transit time beyond the normal rate. Cells depleted of SOD1 display damaged centromeres and mitotic defects but no longer exhibit DNA‐damage‐induced mitotic delay. SOD1 has previously been shown to mediate redox control of phosphatases such as PP2a. In response to DNA damage, we observed elevated phosphorylation of SAC protein BubR1 and the kinetochore protein KNL1. Dephosphorylation of these proteins is required for SAC silencing, and PP2a has previously been implicated in this. Following SOD1 depletion, we observed elevated PP2a activity and decreased phosphorylation of BubR1 and KNL1. We propose that, in response to damage, SOD1 restrains PP2a activity, resulting in elevated BubR1 and KNL1 phosphorylation leading to persistent SAC activation.

## Graphical abstract

DNA damage can slow or delay the cell cycle to allow for DNA repair to occur. However, the mechanism underlying this is unclear. Here, we show that in response to cells entering mitosis with damaged DNA, SOD1 restrains the activity of the phosphatase PP2a activity via oxidation of cysteine residues at PP2a's active site. This leads to a reduction in PP2a activity at the mitotic kinetochore, resulting in elevated phosphorylation of the spindle assembly checkpoint protein BubR1 and kinetochore component KNL1, retention of Plk1, and persistence of the spindle assembly checkpoint. This causes metaphase arrest in cells with damaged DNA.

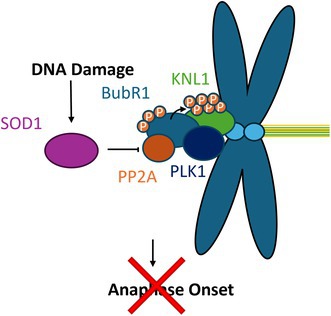

**Abbreviations**
ACA: anti‐centromere antibody
APC/C: anaphase‐promoting complex/cyclosome
Aph: aphidicolin
ATM: ataxia telangiectasia mutated
ATR: ataxia telangiectasia and Rad3 related
BubR1: Bub1‐related kinase 1
Carbo: carboplatin
CDK: cyclin dependent kinase
CENP B: centromere protein B
Chk2: checkpoint protein 2
H_2_O_2_
: hydrogen peroxide
IR: irradiation
KNL1: kinetochore scaffold 1
KO: knock out
Mad2: mitotic arrest deficient 2
MELT: methionine‐glutamine‐leucine‐threonine
pH3: phosphorylated histone H3
Plk1: polo‐like kinase 1
PP1: protein phosphatase 1
PP2a: protein phosphatase 2a
Redox: reduction‐oxidation
SAC: spindle assembly checkpoint
siRNA: small inhibitory RNA
SOD1: superoxide dismutase 1
TMZ: temozolomide
Wee1: G2 checkpoint kinase Wee1
WT: wild‐type
XRCC4: X‐ray repair cross complementing protein 4
γH2AX: phosphorylated histone 2A X

## Introduction

It is vital that cells maintain genomic integrity in order to pass on a faithful copy of their genetic material to the next generation. All the cells in the body are continuously exposed to genotoxic threats, with tens of thousands of DNA‐damaging events occurring in each cell every day. The cellular response to DNA damage involves careful coordination of cell cycle control, DNA repair and programmed cell death in order to maintain genomic integrity.

In response to DNA damage, the phosphatidylinositol 3‐kinase‐related kinases ATM and ATR activate cell cycle checkpoints throughout interphase, resulting in cell cycle arrest at the G1/S and G2/M boundaries and slowing of DNA replication in S phase via inhibition of the cyclin‐dependent kinases (CDKs) [1]. Whilst cell cycle control and activation of DNA repair are well‐characterized in interphase, how and, in fact, whether the cell cycle responds to DNA breaks in mitotic cells remains unclear. Despite published evidence of slowed mitotic transit in response to DNA damage [2, 3, 4, 5], it is generally accepted that there is no DNA damage‐induced checkpoint in mitosis [6]. The reasoning for this is twofold. First, whilst the interphase checkpoints all act through the inhibition of the various CDKs necessary for cell cycle progression [7], there are several mechanisms that prevent this from happening in mitosis; there is little transcription in mitosis, meaning that p21 cannot be induced to inhibit the CDKs. Furthermore, the mitotic kinase Plk1 directly inhibits Wee1 and Claspin, which are required for the inhibitory phosphorylation of CDK1 at Tyrosine 15 [6]. Second, the canonical DNA damage repair pathway is largely inhibited in mitosis [8], which has led to the hypothesis that there is no requirement for a mitotic DNA damage checkpoint. Whilst the signaling cascade that responds to DNA double‐strand breaks in interphase is initiated in mitosis, the cascade has been shown to be attenuated in mitotic cells, resuming in full following the completion of mitosis [8]. However, despite the restriction of the canonical break repair pathways, recent evidence has highlighted break processing in mitotic cells. Broken chromosomes are ‘tethered’ together in mitosis to allow for faithful segregation of fragmented chromosomes [9, 10] and DNA synthesis and recruitment of repair proteins in mitosis have been observed in response to DNA breaks induced in mitotic cells [11, 12, 13].

In 2000, Smits *et al*. demonstrated that mitotic DNA damage inhibits Plk1 in mitosis and significantly delays mitotic exit in U2OS cells [2]. They concluded that Plk1 is an important target of the DNA damage checkpoint leading to cell cycle arrest in mitosis. The mechanism, however, was vague, and Mikhailov *et al*. set out to expand upon this a few years later [3]. They hypothesized that there were three possibilities: (a) DNA damage prevents activation of the APC/C via downregulation of Plk1 activity, (b) DNA damage directly prevents Cyclin A degradation, which is required for metaphase/ anaphase transition and (c) DNA damage does not prolong mitosis via a DNA damage checkpoint but instead through the spindle assembly checkpoint. They demonstrated that whereas extensive DNA damage in mitosis led to metaphase delay, normal spindle formation and cyclin A degradation were observed. Furthermore, the metaphase block was not affected by caffeine, indicating that the metaphase block is ATM independent. They went on to demonstrate that cells blocked in metaphase by DNA damage had at least one Mad2‐positive kinetochore and rapidly exited mitosis upon microinjection of a dominant‐negative Mad2 mutant. They concluded that extensive DNA damage compromises kinetochore function, leading to prolonged activation of the SAC [3].

In this manuscript, we demonstrate that mitotic transit is significantly slowed for up to 16 h post exposure to a range of DNA damaging agents and not just when DNA is damaged directly in mitotic cells. Through an unbiased, high‐throughput siRNA screen, Superoxide dismutase (SOD1) was identified as an essential factor for mitotic delay following exposure to DNA damaging agents. Furthermore, we show that cells with reduced SOD1 levels exhibit higher PP2a activity and reduced BubR1 and KNL1 phosphorylation, indicating a potential mechanism for spindle checkpoint control by SOD1. SOD1 is most known for its role in the conversion of toxic superoxide radicals ($O_{2}^{-}$) to the more stable and less toxic hydrogen peroxide and dioxygen [14]. More recently, SOD1 has also been implicated in the DNA damage response [15, 16] although the mechanism for this is unknown.

## Results

### An siRNA screen for proteins involved in a mitotic DNA damage checkpoint

**Fig. 1:**
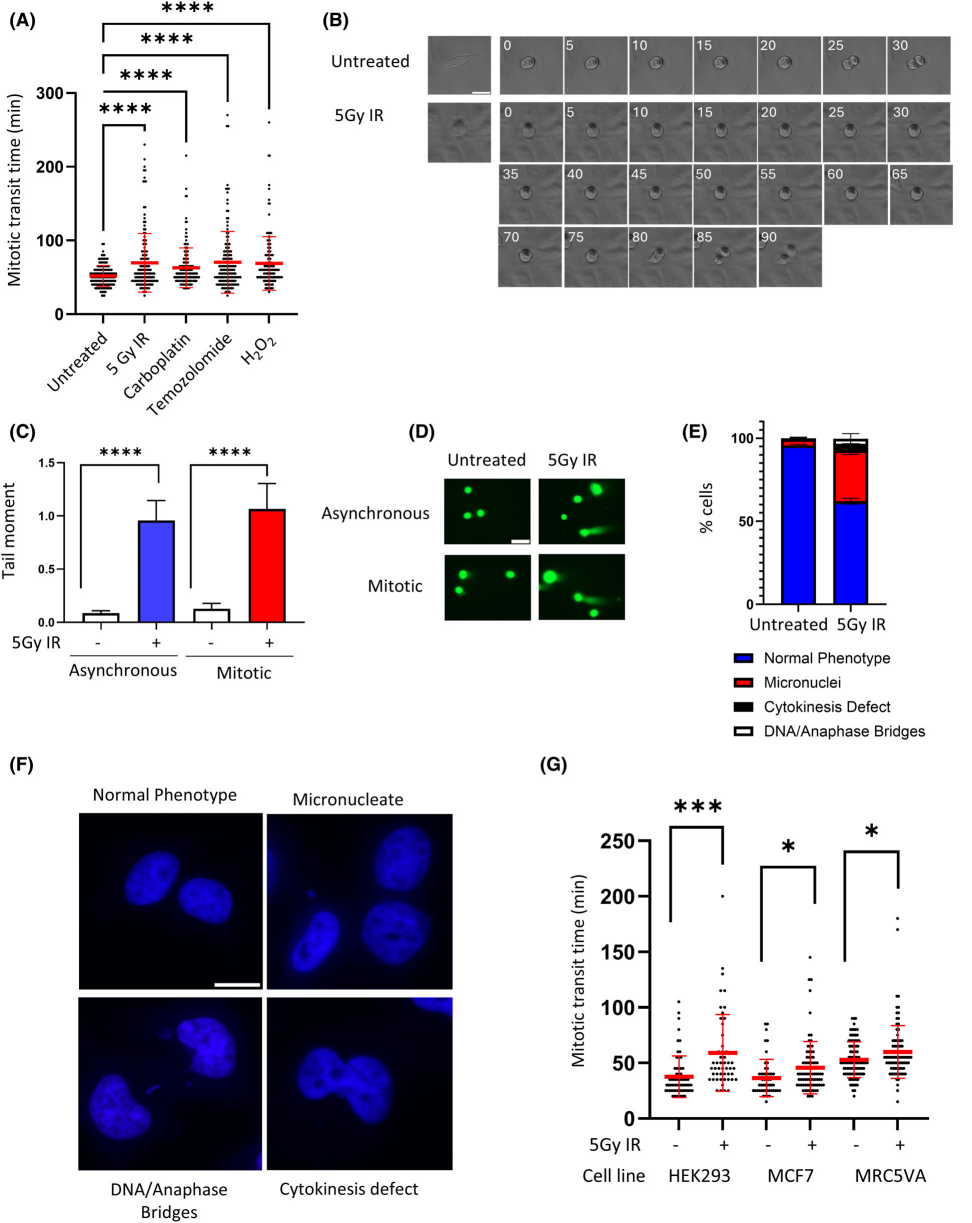
Slowed mitotic transit in response to DNA damage. (A) DNA damaging agents induce mitotic delay in HeLa cells. HeLa cells treated with the indicated agents were viewed by time lapse microscopy. Time in mitosis was scored from time cells rounded up in mitosis to time to cytokinesis. Error bars represent mean ± SEM of three independent experiments (*n* > 150) and were analysed by one way ANOVA with Dunnetts post test. ****P ≤ 0.0001. (B) Images from representative time lapse movies, time indicated in minutes. Scale bar indicates 50 μm. (C) HeLa cells were treated as indicated and then analysed using the neutral comet assay. Red bars represent mean of three independent experiments and datasets were analysed using the students *t*‐test (****P ≤ 0.0001). (D) Representative images from neutral comet assay. Scale bars indicates 100 μm. (E) HeLa cells were fixed 16 h after 5Gy irradiation and analysed by fluorescence microscopy for chromosomal abnormalities. Error bars represent mean ± SD of three biological replicates (*n* > 150). (F) Representative images of (E). Scale bars indicate 50 μm. (G) HEK293, MCF7 and MRC‐5 Cells treated as indicated were viewed by time lapse microscopy. Time in mitosis was scored from time cells rounded up in mitosis to time to cytokinesis. Red bars represent mean of three independent experiments (*n* > 150) and datasets were analysed using a One‐Way ANOVA with Tukey's post‐test. *P ≤ 0.05, ***P ≤ 0.001. Where statistics are not annotated, assume nonsignificance.

To investigate the mitotic cell cycle response to DNA damage, HeLa cells were treated with DNA damaging agents followed by live cell time‐lapse microscopy analysis. We observed that the average time spent in mitosis was significantly increased following the introduction of DNA damaging agents and replication inhibitors (Fig. 1A, Videos S1 and S2). Mitotic transit time was assessed as the duration between mitotic cell rounding and cytokinesis (Fig. 1B). Unlike in previous studies [3], this observed delay was not restricted to cells inflicted with DNA damage whilst in mitosis, as the delay was observed upwards of 16 h post irradiation, indicating that cells can traverse earlier interphase checkpoints and still experience mitotic difficulties. Comet assays on asynchronous vs. mitotic cells (Fig. 1C,D) and visualization of chromosomes by DAPI staining (Fig. 1E,F) revealed that at 16 h post irradiation, mitotic cells had both persistent DNA breaks and chromosomal abnormalities. To investigate whether the mitotic response to DNA damage was cell line specific, three other cell lines were exposed to 5Gy IR and analysed by live cell microscopy for mitotic duration. All cell lines tested exhibited an observable increase in mitotic transit time following exposure to irradiation (Fig. 1G).

**Fig. 2:**
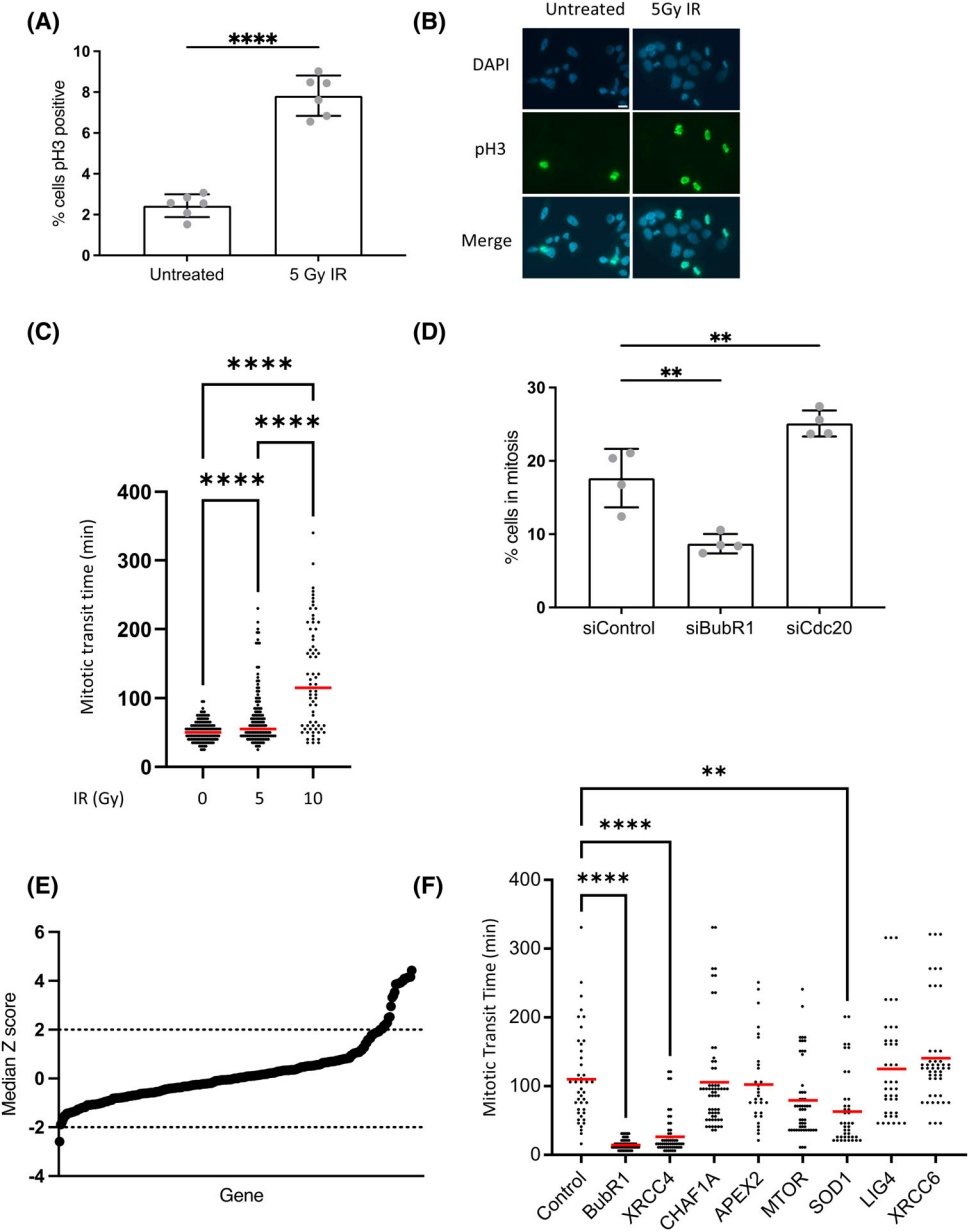
siRNA screen for DNA repair factors involved in DNA damage‐induced mitotic delay. (A) Cells were treated as indicated then harvested and stained for pH3 and propidium iodide prior to analysis via flow cytometry 16 h post irradiation. Mean ± SEM of three independent experiments. Error bars represent standard deviation of six individual repeats and were analysed by one way ANOVA with Dunnetts post test. ****P ≤ 0.0001. (B) Representative images of (A). Scale bars indicate 50 μm. (C) HeLa cells treated as indicated were viewed by time lapse microscopy. Time in mitosis was scored from time cells rounded up in mitosis to time to cytokinesis. Red bars represent mean of three independent experiments (*n* > 150) and were analysed by one way ANOVA with Tukey's post test. ****P ≤ 0.0001. (D) Cells incubated with the indicated siRNAs for 48 h were harvested 16 h post 10Gy irradiation then stained for pH3 and DAPI prior to analysis via fluorescence microscopy. Error bars represent standard deviation of four individual repeats and were analysed by one way ANOVA with Dunnetts post test. **P ≤ 0.01. (E) HeLa cells were plated in 96‐well plates containing the Dharmacon DNA damage siRNA library and incubated for 48 h. Plates were then irradiated (10Gy) and incubated for a further 16 h prior to staining for pH3 and PI. Analysis was performed via high‐throughput microscopy. Graph depicting rank ordered mean Z‐scores from siRNA screen. Data points represent mean Z scores from five biological replicates (*n* > 25). (F) HeLa cells incubated for 48 h with the indicated siRNAs prior to exposure to ionizing radiation (10Gy) were viewed by time lapse microscopy. Time in mitosis was scored from time cells rounded up in mitosis to time to cytokinesis. Red bars represent mean of three independent experiments (*n* > 150) and were analysed by one way ANOVA with Dunnetts post test. **P ≤ 0.01, ****P ≤ 0.0001.

DNA damage induced mitotic delay was also observable as an increase in cells expressing the mitotic marker protein phosphorylated Histone‐H3, 16 h after IR treatment (Fig. 2A,B) allowing for the development of a high‐throughput siRNA screen to uncover members of the DNA damage response involved in a potential mitotic DNA damage checkpoint. We found that increasing the dose of radiation further increased the mitotic duration (Fig. 2C) and therefore, 10Gy IR was used for the screen in order to enhance the effects. The screening conditions were optimized using siRNA to the spindle assembly checkpoint protein, BUBR1, to reduce the number of cells in mitosis and siRNA to anaphase‐promoting complex protein, CDC20, to increase the number of cells in mitosis (Fig. 2D). Whilst the percentage of mitotic cells determined by the automated software was different from the levels previously detected by flow cytometry, we were satisfied that the decrease in the mitotic population after siBubR1 and the increase upon incubation with siCdc20 meant that the methodology was sufficient for the screen. The screen was performed five times, and data were rank‐filtered by the mean ordered Z‐score (Fig. 2E). A stringent Z‐score cut‐off of 2 was applied, identifying 11 siRNAs that significantly reduced the mitotic population. A secondary screen using live cell imaging to assess mitotic transit time after 10Gy IR was conducted to specifically identify siRNAs that reduced the number of cells in mitosis due to a reduction in mitotic transit time (Fig. 2F). Four siRNAs were excluded at this point as they had an impact on interphase progression, meaning no cells progressed to mitosis. The secondary screen revealed that siRNA against the nonhomologous end joining protein, XRCC4, and the reactive oxygen species reducing protein, SOD1, both significantly reduced mitotic transit time.

### The impact of screen hits on the canonical SAC

**Fig. 3:**
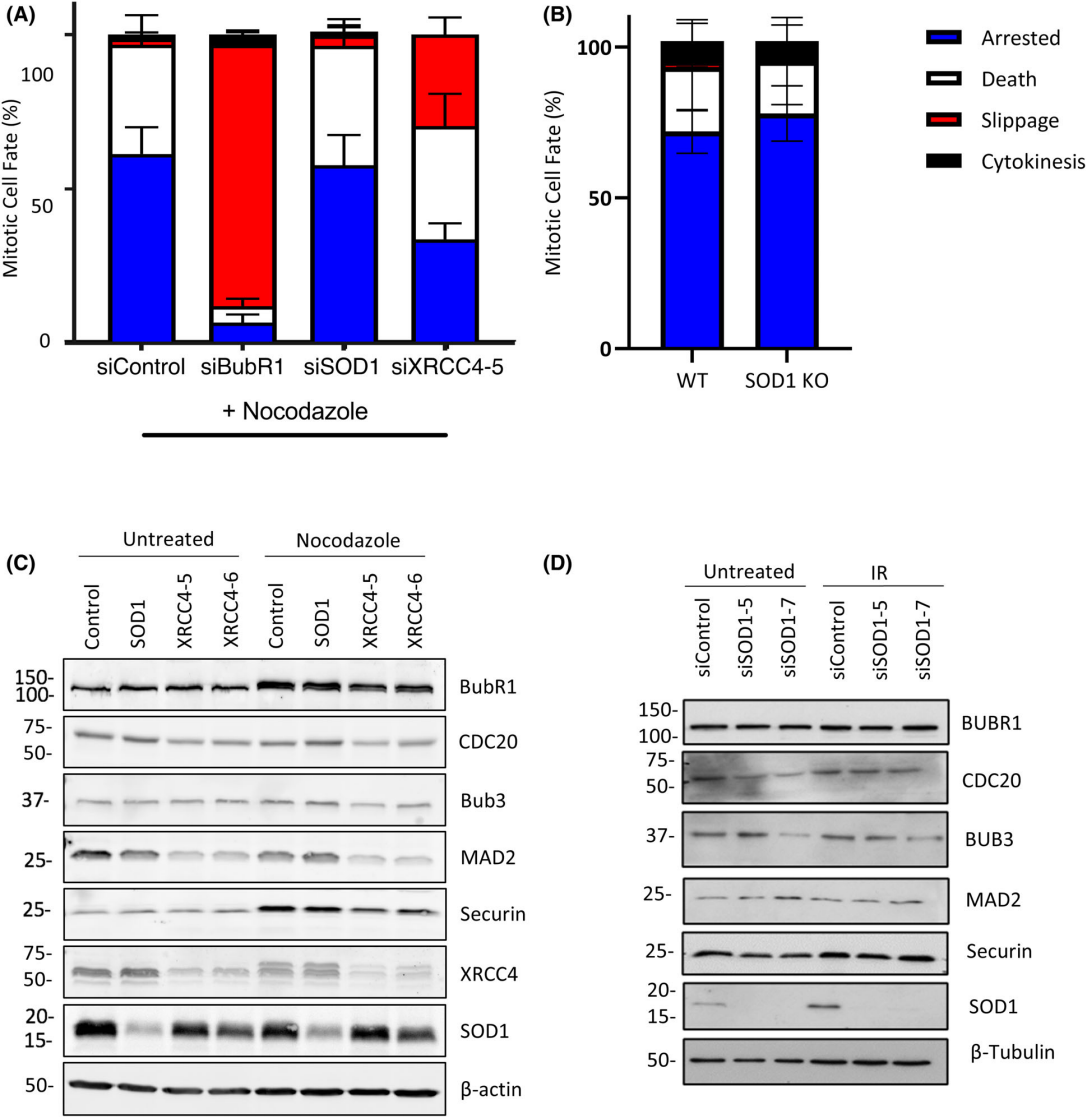
siRNA‐mediated depletion of SOD1 does not impact the canonical spindle assembly checkpoint. (A) Cells were transfected with indicated siRNAs for 72 h. Cells were visualized by live cell imaging following treatment with 200 ng·mL^−1^ Nocodazole for the remaining 16 h and cell fate was scored. Error bars represent SEM of three individual repeats (*n* = 150). (B) HeLa‐SOD1^WT^ and HeLa‐SOD1^KO^ cells were treated with 20 ng·mL^−1^ Nocodazole and visualized by live cell imaging for 16 h and cell fate was scored. Error bars represent SEM of three individual repeats (*n* = 150). (C) HeLa cells were incubated with the indicated siRNAs for 48 h prior to treatment. 16 h post nocodazole cells were lysed and proteins were analysed by western blot. Representative data of *n* = 3. (D) HeLa cells were incubated with the indicated siRNAs for 48 h prior to treatment. 16 h post irradiation, cells were lysed and proteins were analysed by western blot. Representative data of *n* = 3.

As the key regulator of cell cycle progression in mitosis is the spindle assembly checkpoint (SAC) which assesses kinetochore attachment prior to mitosis, we investigated whether XRCC4 and SOD1 had an impact on the canonical SAC. Cells were treated with the indicated siRNAs for 48 h and then exposed to the spindle poison nocodazole (200 μg·mL^−1^) prior to the initiation of live cell imaging. In the population treated with the control siRNA, nocodazole induced either mitotic arrest (Video S3) or death in mitosis (Video S4) in almost 100% of cells, whereas the vast majority of cells treated with siRNA to BUBR1 underwent a process known as mitotic slippage (Video S5), whereby cells exit mitosis without undergoing cytokinesis. siRNA to SOD1 was found to have similar effects on mitosis in the presence of nocodazole as in the siControl, with the vast majority of cells undergoing either mitotic arrest or death in mitosis (Fig. 3A). To exclude the possibility that the phenotype was caused by high concentrations of nocodazole, we exposed paired HeLa SOD1 wild‐type (HeLa‐SOD1^WT^) and SOD1 knockout (HeLa‐SOD1^KO^) cells to 10‐fold less nocodazole and found similar effects remaining between them (Fig. 3B). siRNA‐mediated depletion of XRCC4, however, resulted in a similar effect to depletion of BubR1, with the majority of cells undergoing mitotic slippage (Fig. 3A). Western blotting revealed no changes in expression of the key SAC proteins following siRNA‐mediated depletion of SOD1 in response to either nocodazole (Fig. 3C) or IR (Fig. 3D) exposure; however, following depletion of XRCC4, marked depletion of Mad2 and other Mitotic Checkpoint Complex proteins was observable (Fig. 3C). Taken together, this indicates that whilst siRNA‐mediated depletion of XRCC4 impacts the canonical SAC, the impact of SOD1 on mitosis is through another level of signaling.

There have been reports of SAC proteins, particularly Mad2, being sensitive to siRNA duplexes with no sequence homology [17]. We therefore concluded that the impact of siRNA‐mediated depletion of XRCC4 on the mitotic response to nocodazole was likely due to off‐target effects of the siRNA duplexes on MAD2 and did not pursue this further.

### SOD1 is required for DNA damage induced mitotic arrest

**Fig. 4:**
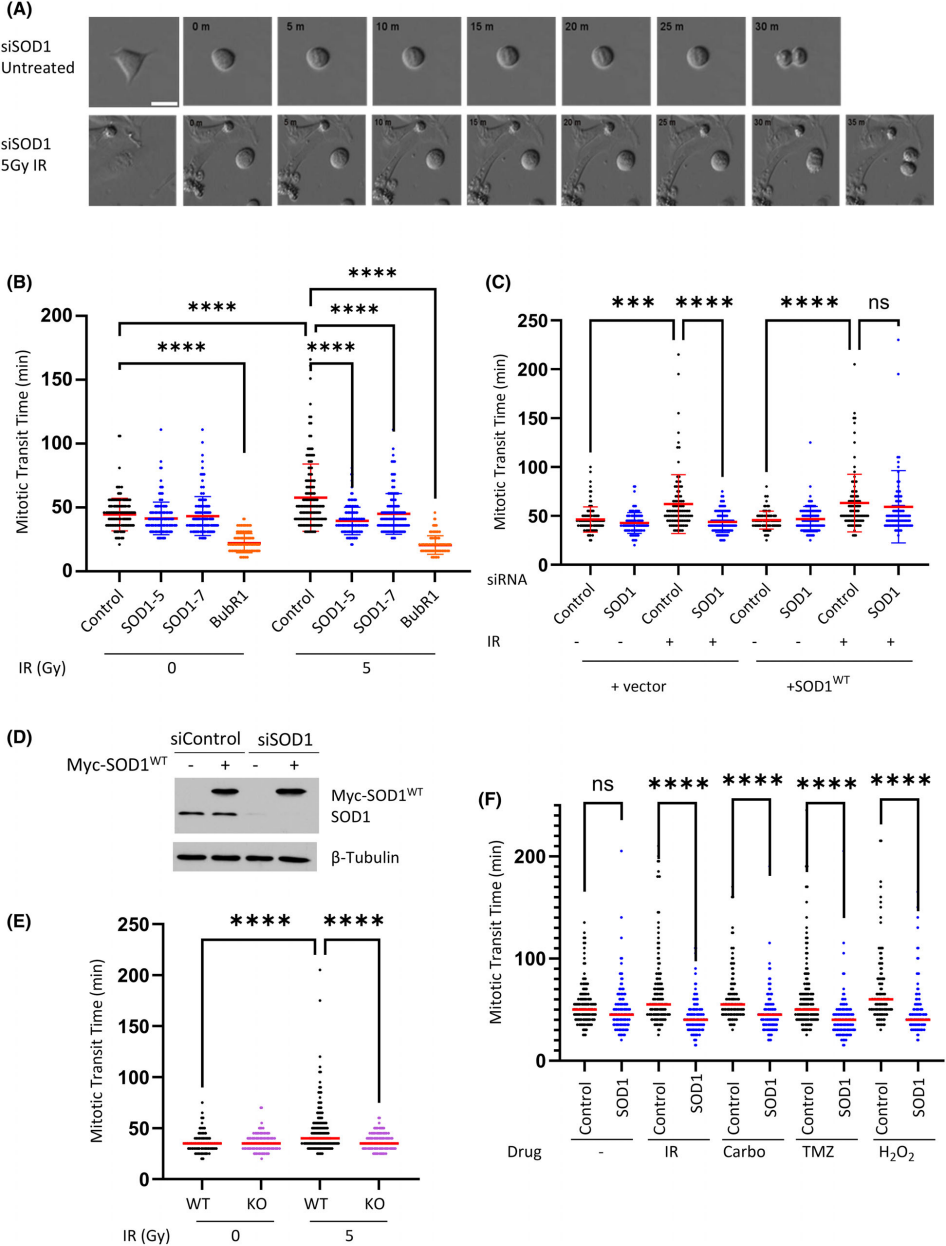
Slowed mitotic transit induced by DNA damage is SOD1‐dependent. (A) Images from representative time lapse movies of siSOD1 cells. Scale bars indicate 50 μm. (B), time indicated in minutes. (B) HeLa cells incubated with the indicated siRNA were viewed by time lapse microscopy with and without irradiation. Mean ± SEM of three independent experiments (*n* > 150) and were analysed by one way ANOVA with Dunnetts post test. ****P ≤ 0.0001. (C) HeLa cells incubated with the indicated siRNA and cDNA were viewed by time lapse microscopy with and without irradiation. Mean ± SEM of three independent experiments (*n* > 150) and were analysed by one way ANOVA with Dunnetts post test. ***P < 0.001, ****P ≤ 0.0001. (D) Western blotting images from (C). (E) Paired HeLa‐SOD1^WT^ and HeLa‐SOD^KO^ cells were viewed by time lapse microscopy with and without irradiation. Red bars represent mean of three independent experiments (*n* > 150) and datasets were analysed by one way ANOVA with Dunnetts post test. ****P ≤ 0.0001. (F) HeLa cells incubated with the indicated siRNA and cDNA were viewed by time lapse microscopy with and without treatment as indicated. Red bars represent mean of three independent experiments (*n* > 150) and datasets were analysed by one way ANOVA with Dunnetts post test. ****P ≤ 0.0001.

As with nocodazole treatment (Fig. 3A), and in contrast to BubR1 depletion, SOD1 depletion was found to have no effect on mitotic progression in the absence of DNA damage (Fig. 4A,B). Following exposure with 5Gy IR, the mean time spent in mitosis increased from 52.56 to 62.47 min in the cells treated with siControl; however, this was not observed in cells treated with two independent siRNAs to SOD1 (Fig. 4A,B, Videos S6 and S7). Ectopic overexpression of Myc‐tagged SOD1 was sufficient to fully restore mitotic delay in response to DNA damage, demonstrating that SOD1 is responsible for this effect (Fig. 4C,D). This data was further corroborated by exposing the paired HeLa‐SOD1^WT^ and HeLa‐SOD1^KO^ cells to 5Gy IR, whereby the wild‐type cells exhibited arrest, whereas the SOD1 knockout cells did not (Fig. 4E). SOD1 was also found to be responsible for arrest in response to other DNA damaging agents tested (Fig. 4F). Taken together with Fig. 3, this demonstrates that the impact of SOD1 on mitotic progression is specific to DNA damage.

**Fig. 5:**
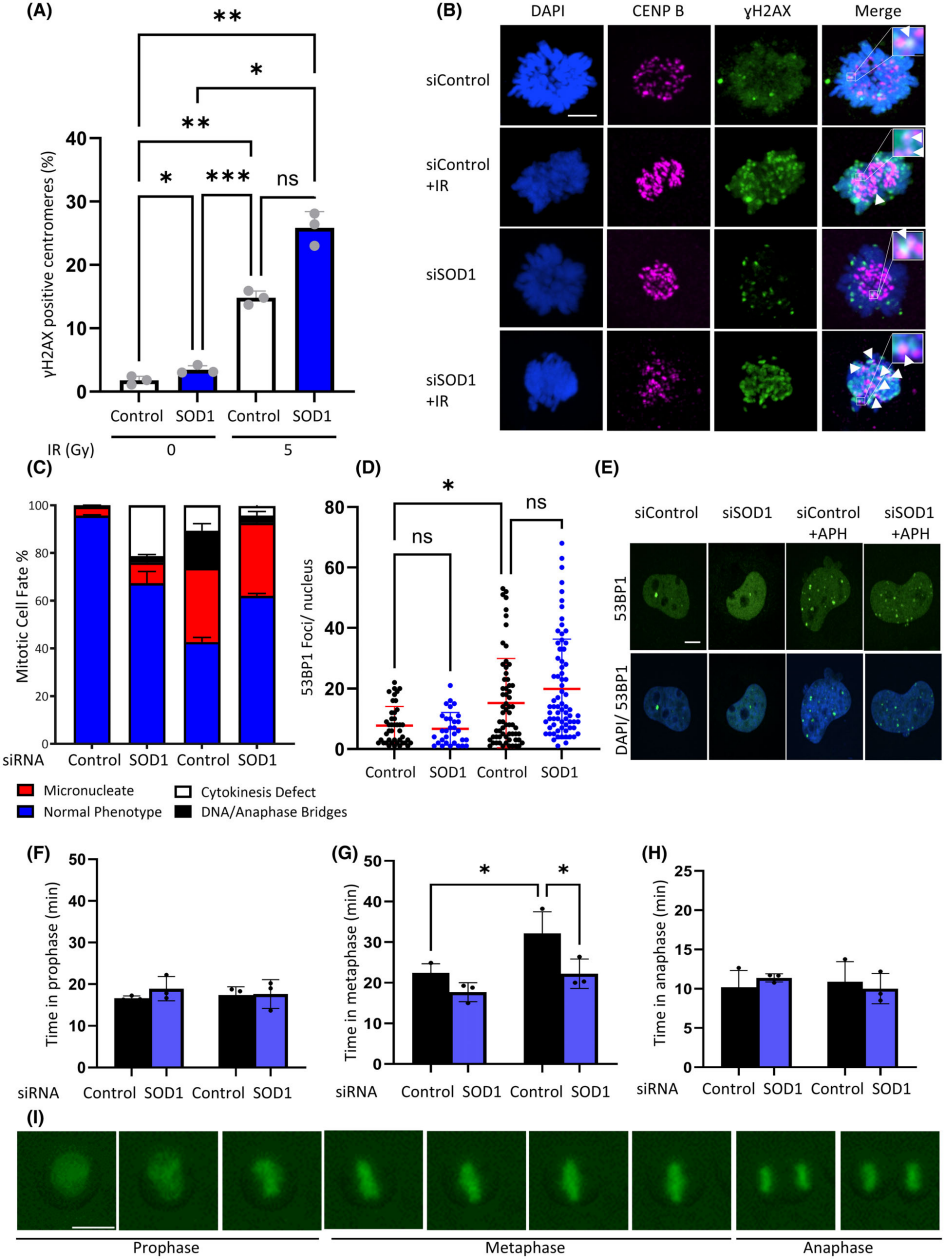
SOD1 depletion abolishes DNA damage‐induced metaphase arrest and results in elevated DNA damage and chromosomal abnormalities. (A) HeLa cells plated on coverslips were transfected with the indicated siRNAs for 72 h and harvested 16 h post 5Gy irradiation. Slides were stained for the proteins indicated and γH2AX‐positive centromeres were scored. Errors bars represent mean ± SEM of three individual repeats (*n* = 150). Results analysed by one way ANOVA with the Tukey post test. *P ≤ 0.05, **P ≤ 0.001, ***P ≤ 0.0001. (B) Representative images of (A). White scale bar indicates 5 μm. Black scale bars indicate 500 nm. (C) HeLa cells, transfected for 48 h with the indicated siRNAs were fixed 16 h after 5Gy irradiation and analysed by fluorescence microscopy for chromosomal abnormalities. Error bars represent mean ± SD of three independent experiments (*n* > 150). (D) HeLa cells were transfected for 48 h with the indicated siRNAs. Mitotic cells were then collected 16 h after incubation with 0.4 μm aphidicolin, incubated for 150 min to allow to progress to G1 and stained for 53BP1 foci. The images were analysed using image j. Error bars represent mean ± SD of three independent experiments (*n* > 150) Results analysed by one way ANOVA with the Tukey post test. *P ≤ 0.05. (E) Representative images of (D). Scale bar indicates 5 μm. (F–H) HeLa cells expressing the H2B‐GFP construct were incubated with the indicated siRNA and viewed by time lapse microscopy with and without irradiation (5Gy). Mean ± SEM of three independent experiments (*n* > 150) and were analysed by one way ANOVA with Dunnetts post test. *P < 0.05, **P ≤ 0.01. (I) Representative images of (F–H). Scale bar indicates 50 μm.

Previous studies have suggested that DNA damage induced in mitosis results in metaphase arrest through persistent SAC activation resulting from unattached spindles due to direct damage of kinetochores [3, 13]. Whilst we also saw elevated DNA breaks specifically at kinetochores in response to DNA damage, this was also the case in the absence of SOD1 (Fig. 5A,B). Moreover, in the absence of SOD1, there was a significant increase in various mitotic defects compared with the control in the presence of DNA damage (Fig. 5C). This mitotic dysfunction correlates with elevated 53BP1 foci in the following G1 compared to the control cells (Fig. 5D,E), suggesting these cells prioritize completion of mitosis for repair in the following cell cycle. The use of a GFP tagged H2B protein construct allowed for greater depth analysis of the DNA and mitotic progression in the presence of DNA damage. This revealed that SOD1‐dependent DNA damage induced mitotic arrest from damage induced outside mitosis also occurs in metaphase (Fig. 5F–I), indicating involvement of the spindle assembly checkpoint. However, with depletion of SOD1, an extra level of signaling overrides the activation of the spindle assembly checkpoint, leading to mitotic progression despite DNA damage.

### SOD1 depletion leads to increased PP2a activity and reduced BubR1 and KNL1 phosphorylation in response to DNA damage

The cell cycle is largely regulated by phosphorylation and dephosphorylation of protein cascades, dependent on kinases and phosphatases. The key phosphatase regulating the spindle assembly checkpoint and mitotic exit is PP2a [18, 19]. Previous studies have shown that phosphatases are sensitive to redox inhibition; H_2_O_2_ can directly oxidize cysteines in the active site of PP2a, leading to a reduction in activity [20]. This oxidation and subsequent reduction in PP2a activity were found to be SOD1 dependent [21] either due to the role of SOD1 in producing H_2_O_2_ from superoxide radicals [21] or through direct oxidation of thiol groups by SOD1 [22]. Recent studies have identified a proteome‐wide thiol redoxome of SOD1‐derived H_2_O_2_ vital for defense against antioxidants [23].

**Fig. 6:**
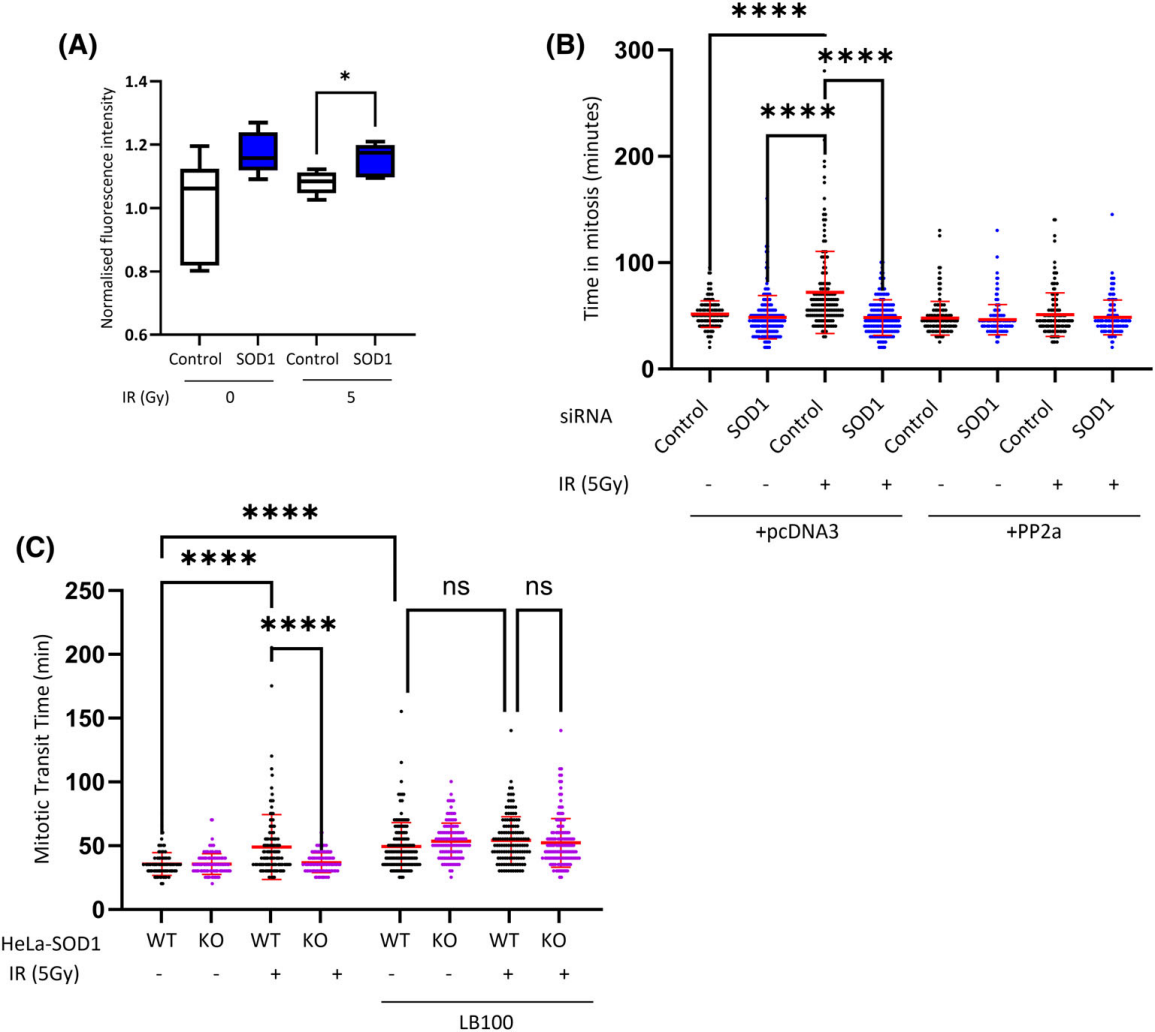
SOD1 represses PP2a activity to control mitotic progression. (A) Cells were incubated with the indicated siRNAs for 72 h and analysed using PP2a activity assay (Sigma Aldrich). Mean ± 5 individual repeats. Each dataset normalized to the untreated control. Analysed by unpaired *t*‐test. * = *P* ≤ 0.05. (B) HeLa cells incubated with the indicated siRNA and cDNA were viewed by time lapse microscopy with and without irradiation. Mean ± SEM of three independent experiments (*n* > 150) and were analysed by one way ANOVA with Dunnetts post test. ****P ≤ 0.0001. (C) HeLa‐SOD1^WT^ and HeLa‐SOD1^KO^ were viewed by time lapse microscopy with and without irradiation and PP2a inhibitor LB100. Mean ± SEM of three independent experiments (*n* > 150) and were analysed by one way ANOVA with Dunnetts post test. ****P ≤ 0.0001.

We found that, consistent with the published literature [20], SOD1 knockdown resulted in elevated PP2a activity (Fig. 6A). In order to test whether PP2a is responsible for mitotic delay in the presence of DNA damage, we assessed mitotic transit time after IR treatment following PP2a overexpression (Fig. 6B) and inhibition (Fig. 6C). We found that inhibition of PP2a activity via LB100 was sufficient to induce mitotic delay independently of DNA damage or SOD1 status and that ectopic overexpression of PP2a completely prevents mitotic delay in the presence of DNA damage.

**Fig. 7:**
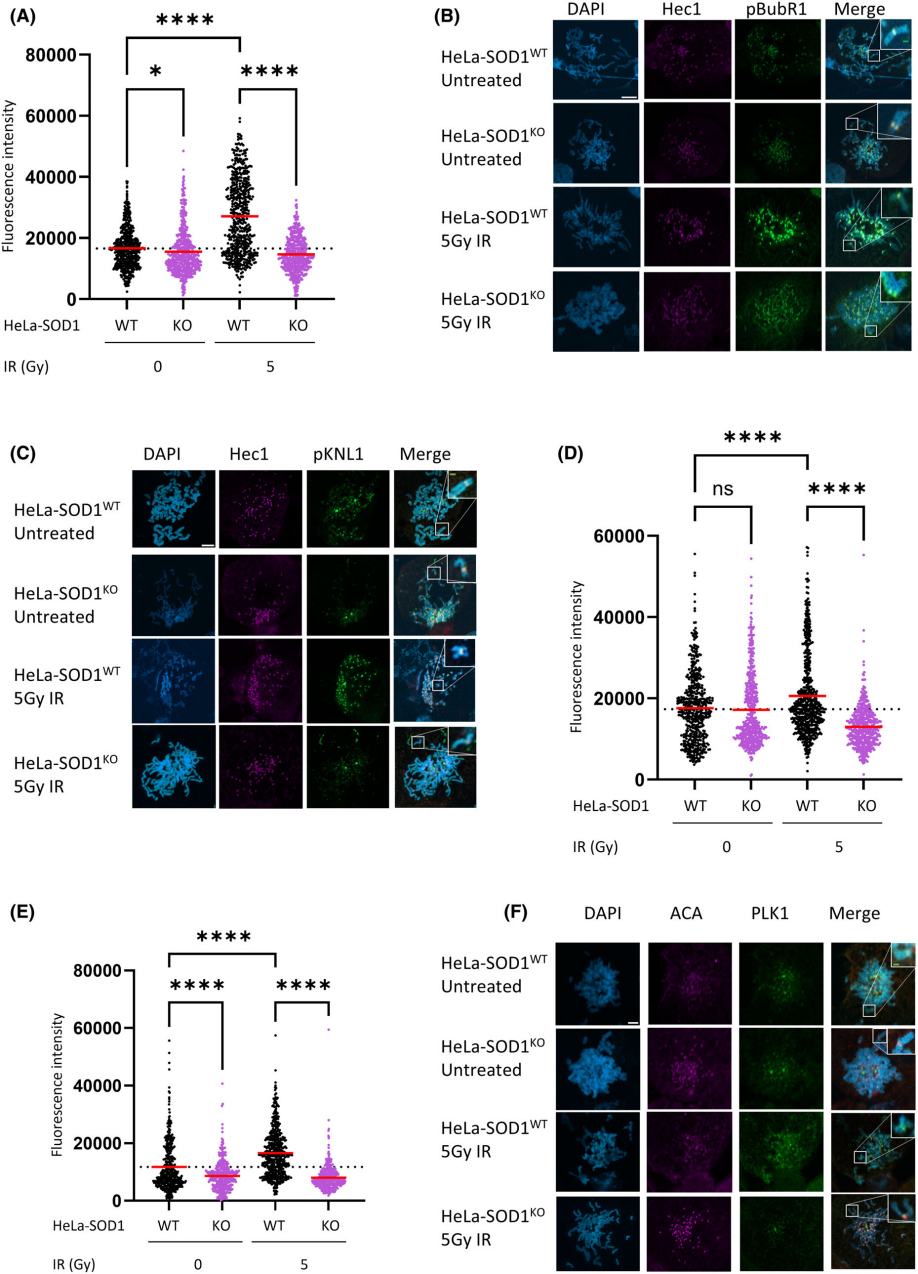
SOD1 knockout results in loss of DNA damage‐induced phosphorylation of SAC effectors. (A) HeLa‐SOD1^WT^ and HeLa‐SOD1^KO^ cells were harvested 16 h post 5Gy irradiation and stained for pBubR1 and Hec1, and were viewed by fluorescence microscopy. Images were analysed for fluorescent intensity of pBubR1 at Hec1 sites using image j. Red line dictates mean of three independent experiments (*n* > 150) and were analysed by one way ANOVA with Dunnetts post test. *P < 0.01. ****P < 0.0001. (B) Representative images of (A). White scale bars indicate 5 μm, Green scale bars indicate 500 nm. (C) Representative images of (D). Scale bars indicate 5 μm. (D) HeLa‐SOD1^WT^ and HeLa‐SOD1^KO^ were harvested 16 h post 5Gy irradiation and stained for pKNL1 and Hec1 were viewed by fluorescence microscopy. Images were analysed for fluorescent intensity of pKNL1 at Hec1 sites using image j. Red line dictates mean of three independent experiments (*n* > 150) and were analysed by one way ANOVA with Dunnetts post test. ****P < 0.01. (E) HeLa‐SOD1^WT^ and HeLa‐SOD1^KO^ were harvested 16 h post 5Gy irradiation and stained for Plk1 and ACA were viewed by fluorescence microscopy. Images were analysed for fluorescent intensity of PLK1 at ACA sites using image j. Red line dictates mean of three independent experiments (*n* > 150) and were analysed by one way ANOVA with Dunnetts post test. ****P < 0.01. (F) Representative images of (E). Scale bars indicate 5 μm.

Together with its B56 regulatory subunit, PP2a interacts with the spindle assembly checkpoint protein BubR1 at the Serine 670 (S670) residue in mitosis [24, 25]. Both improper phosphorylation and dephosphorylation of BubR1 at S670 have been shown to result in mitotic delay [26]. This localized phosphatase activity at the kinetochore is required to silence the SAC through removal of Plk1 leading to dephosphorylation of the kinetochore protein KNL1 [27]. Here, we show increased BubR1 phosphorylation at S670 (Fig. 7A,B) and KNL1 phosphorylation on the MELTS motif (Fig. 7C,D) at mitotic kinetochores in response to DNA damage, both of which are significantly reduced in cells depleted of SOD1. Furthermore, we see corresponding increased levels of Plk1 at kinetochores in response to IR (Fig. 7E,F) indicating that the increased phosphorylation of BubR1 and KNL1 results in a stronger and prolonged SAC response.

We propose that in response to DNA damage, SOD1 restrains PP2a activity, leading to reduced PP2a at kinetochores. This results in elevated BubR1 and KNL1 phosphorylation and Plk1 recruitment, leading to prolonged activation of the SAC.

## Discussion

Following the observation in 2000 that DNA damage leads to Plk1 inhibition in mitosis [2], Mikhailov *et al*. set out to assess the mitotic response to DNA damage in detail. They observed that most previous studies had used mitotic spindle poisons in their assays, which complicates events by prolonging the spindle assembly checkpoint regardless of DNA damage. Their assays focussed on inducing DNA damage directly in mitotic cells. They observed that only major DNA damage was capable of instigating a prolonged mitosis, and they found no standard DNA checkpoint inhibitors were able to override this arrest, leading them to conclude that DNA damage induced metaphase arrest is not a DNA damage checkpoint response. The only thing they found able to prevent arrest was a dominant negative Mad2 construct, leading them to conclude that cells were arrested by the canonical spindle checkpoint in response to DNA damage, despite normal spindles [3]. More recent studies have also observed that mitotic DNA synthesis in response to mitotic DNA breaks induces mitotic arrest, which is dependent on the spindle checkpoint [13].

Whilst our experiments have a key difference to those by Mikhailov *et al*., in that we study cells which have entered mitosis following exposure to damage in interphase as opposed to directly inducing damage in mitosis, we also observed that DNA damage‐induced mitotic arrest occurs at metaphase indicating involvement of the spindle checkpoint. Furthermore, we found that blocking the spindle checkpoint, through depletion of spindle checkpoint protein BubR1, could override DNA damage‐induced metaphase arrest; however, we found that the rapid mitotic transit time in response to BubR1 depletion occurred regardless of DNA damage. In contrast, depletion of SOD1 has no impact on normal mitosis or on cells exhibiting mitotic arrest due to spindle disruption; however, it abolishes mitotic delay in response to DNA damage. We also found that whilst DNA breaks at kinetochores were evident in response to DNA damage, which could lead to the kinetochore dysfunction proposed by Mikhailov *et al*., these were still apparent in cells depleted for SOD1. Taken together, these data indicate that mitotic arrest in response to DNA damage requires an extra level of signaling control outside of the canonical SAC.

SOD1 is most known for its role in the conversion of toxic superoxide radicals ($O_{2}^{-}$) to hydrogen peroxide and dioxygen [14]. More recently, SOD1 has been implicated in the DNA damage response with elevated levels of DNA damage seen in SOD1 mutant cells [15] and overexpression of SOD1 leading to activation of the DDR in SOD1 mutant cells [16]. Loss of SOD1 has also been shown to confer sensitivity to DNA damaging agents and lead to downregulation of the ATM pathway in yeast [28]. In addition, SOD1 has also been implicated in regulation of gene expression. SOD1 was found to be activated by DDR proteins ATM and Chk2 [29, 30, 31] and in turn, acted as a transcription factor to initiate gene expression of redox and DNA damage related proteins [29].

**Fig. 8:**
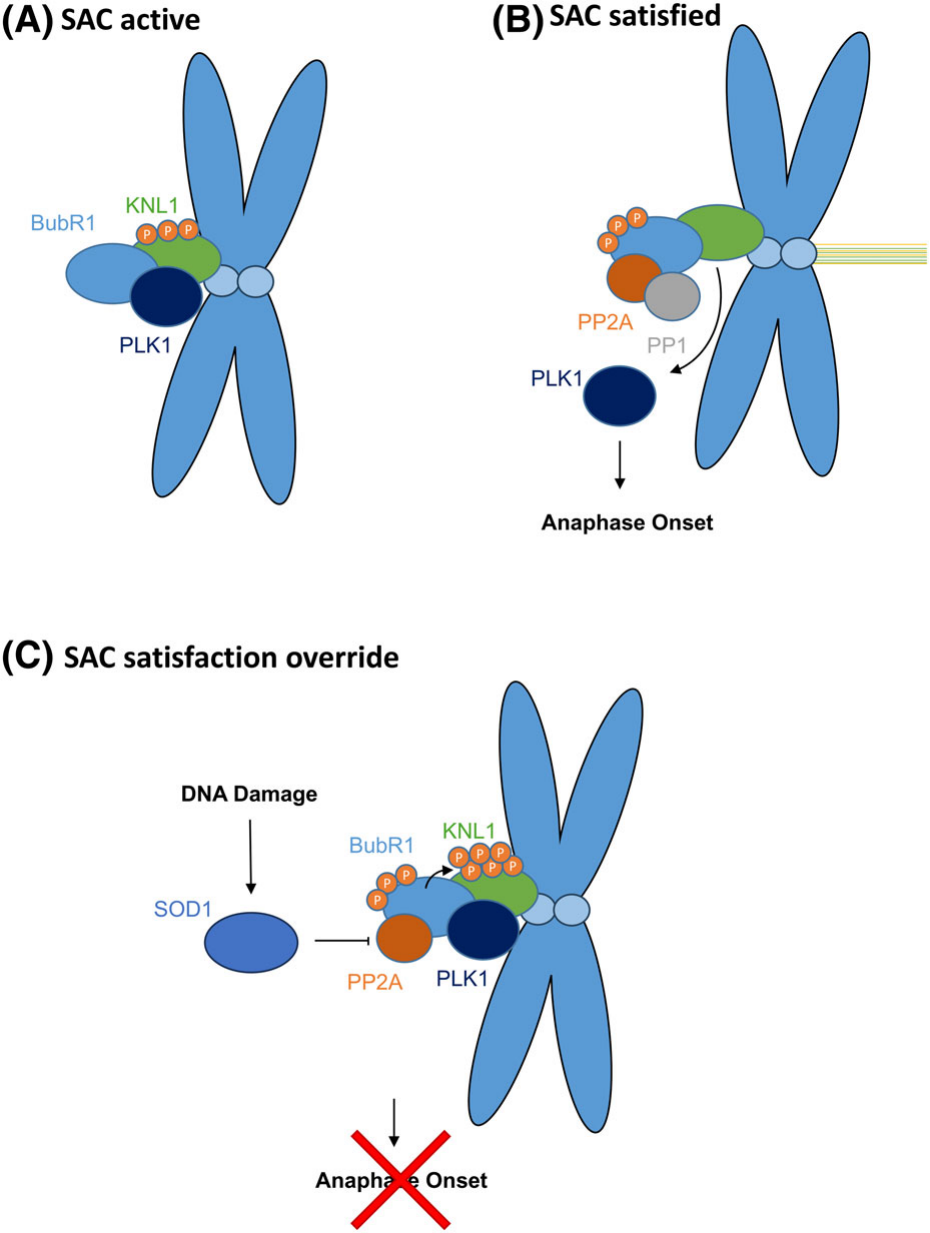
Proposed mechanism for DNA damage‐induced metaphase arrest. (A) Upon entry to mitosis, BubR1 is recruited to the kinetochore and along with KNL1, sequesters Plk1, preventing anaphase onset. (B) Following proper kinetochore attachment, BubR1 phosphorylation on S670 results in recruitment of PP2a which then recruits PP1 for the dephosphorylation of BubR1 and KNL1‐MELTs, resulting in Plk1 release from the kinetochore, allowing for anaphase progression. (C) In response to DNA damage, SOD1 restrains PP2a activity, leading to elevated BubR1 and KNL1 phosphorylation, and retention of Plk1 resulting in metaphase arrest.

SOD1 has previously been shown to regulate phosphatases, including PP2a, through the production of H_2_O_2_ leading to oxidation of cysteine residues in the active site [20, 21] to control growth factor signaling [21] and apoptosis [32]. Our data was consistent with this observation as we demonstrated elevated PP2a activity in cells depleted of SOD1. Whilst the evidence for PP2a involvement in mitotic exit is quite well established, there is conflicting data around the involvement of PP2a in the spindle checkpoint. siRNA to either PP2a or its binding partner B56 has been shown to delay spindle assembly and SAC silencing [19, 33]; however, other studies have found that dephosphorylation of CDK1 substrates required for mitotic progression is unaffected by PP2a inhibition [34], and PP2a inhibitors have no impact on progression after nocodazole or in MCC maintenance [35]. More recent studies have shed light on this complex signaling, demonstrating that specifically, outer kinetochore localized PP2a‐B56 is required for SAC silencing. In response to BUBR1 phosphorylation at S670, BUBR1 recruits Plk1 to phosphorylate the MELT domain on KNL1 to maintain SAC signaling; however, it also recruits PP2a. PP2a then recruits PP1 to dephosphorylate BUBR1 at serine 670, which in a negative feedback loop leads to loss of PP2a at kinetochores [33]. PP2a recruitment of PP1 also leads to the dephosphorylation of KNL1 on the MELTS motif, which results in the removal of Plk1 from the kinetochore and the SAC being silenced so mitosis can progress [27]. This complex system of positive and negative feedback loops must require further regulation so the balance can be tipped one way or the other to allow for arrest or progression. Our data suggest that in response to DNA damage or ROS, SOD1 restrains activity of PP2a, resulting in elevated BubR1 phosphorylation and therefore elevated KNL1 pMELT phosphorylation leading to prolonged SAC activation (Fig. 8). Thus far, our studies have not detected SOD1 at kinetochores, meaning it is unclear whether SOD1 mediated restraint of PP2a is localized or cell‐wide. Overall, we have identified a novel signaling pathway in mitosis which occurs in response to DNA damage. This pathway adds a level of control over the standard spindle assembly checkpoint which has no impact on the SAC under normal conditions but can arrest cells with DNA damage in metaphase to allow for DNA break processing and repair.

## Materials and methods

### Cell culture and reagents

HeLa (RRID: CVCL_0030), MCF7 (RRID: CVCL_0031), HEK293 (RRID: CVCL_0045) and MRC‐5 (RRID: CVCL_0440) cells (ATCC, Manassas, VA, USA) were cultured as previously described [5]. Paired HeLa‐SOD1^WT^ and HeLa‐SOD1^KO^ were a kind gift of Dr Carl LaFlamme (Structural Genomics Consortium, Montreal Neurological Institute, Montreal, Canada). All experiments were performed in mycoplasma‐free cells. Irradiation was carried out using the CIB/IBL 437 Cesium‐137 irradiator. Where indicated, cells were treated with Carboplatin (Sigma‐Aldrich, Burlington, MA, USA), LB100 (Stratech Scientific, Ely, UK), Temozolomide (Sigma‐Aldrich), H_2_O_2_ (EMD Millipore, Darmstadt, Germany), and aphidicolin (Santa Cruz, Dallas, TX, USA) at indicated concentrations. Antibodies against SOD1, BUBR1, Phospho‐BUBR1 (S670), 53BP1, MAD2, pH3, HEC1/HEC, and Securin were obtained from Abcam (Cambridge, UK); BUB3, CDC20, Phospho‐KNL1 (Thr943/Thr1155) and Myc‐Tag from Cell Signaling (Danvers, MA, USA); Actin, PLK, and CENP B from Santa Cruz; XRCC4 from Proteintech (San Diego, CA, USA); γH2AX from Novus Bio (Littleton, CO, USA); and β‐Tubulin from Sigma‐Aldrich. Appropriate secondary antibodies conjugated to horseradish peroxidase (Agilent DAKO, Santa Clara, CA, USA) were used for the western blotting experiments, and Alexa‐Fluor 488 and Alexa‐Fluor 594 secondary antibodies (Invitrogen, Waltham, MA, USA) were used for immunofluorescence.

### Flow cytometry

Cells were fixed in 70% ethanol prior to staining. Following PBS washes to remove ethanol, cells were permeabilized by incubation in Flow Buffer 1 (0.5% BSA, 0.25% Triton‐X). Flow Buffer 1 was removed by centrifugation, and the cells were incubated in pH3 antibody (EMD Millipore, 3018868) diluted 1 : 100 in Flow Buffer 1 for 1 h and 30 min at room temperature. Cells were washed 3 times in Flow Buffer 2 (0.25% Triton‐X) then resuspended in 100 μL of the appropriate FITC secondary antibody, diluted 1 : 100 in Flow Buffer 1, and incubated for 30 min at room temperature in the dark. The cell pellets were resuspended in 400 μL PI (10 μg·μL^−1^ stock solution in PBS) containing RNAse A (80 μg·mL^−1^) and incubated for at least 30 min at 4 °C until processing.

The samples were processed on a FACSCalibur (BD Sciences, Franklin Lakes, NJ, USA) and analysed using flowjo.

### RNAi and DNA transfection

siRNA transfections were performed using Dharmafect 1 siRNA transfection reagent (Horizon, Cambridge, UK), DNA transfections using Lipofectamine 2000 (Thermo‐Fisher Scientific, Waltham, MA, USA) and siRNA/DNA cotransfections using Dharmafect Duo (Horizon) according to the manufacturer's instructions. Cells were treated 48 h post transfection. siControl, siBUBR1, siCHAF1A, siAPEX2, siXRCC4, siMTOR, siLIG4, siXRCC6, siSOD1 and siGenome SMARTpool siRNA pools were from Horizon Discovery.

Single siRNA sequences used were:

siControl: (UAAUGUAUUGGAACGCAUA)TT

siBUBR1: (GATTTAGCACATTTACTAT)TT

siSOD1‐5: (UCGUUUGGCUUGUGGUGUA)TT

siSOD1‐7: (GUGCAGGGCAUCAUCAAUU)TT

siXRCC4‐5: (UGACCGAGAUCCAGUCUAU)TT

siXRCC4‐6: (AACCCAGUAUACCCCAUU)TT

PP2A‐Myc‐DDK pCMV6 was from Origene (NM_002715, RC201334, Herford, Germany). SOD1 optimized construct was custom made by Eurofins (Luxembourg City, Luxembourg) and subcloned into pCMV6 with a Myc‐DDK tag.

### siRNA screen

HeLa cells (z‐prime ≥ 0.5) were reverse‐transfected in 384 plates using Dharmafect 1 and 50 nm each 3× siRNA pool targeting one of 240 human DNA repair genes (Dharmacon Human siGenome siRNA library DNA damage response), negative (four individual nontargeting siRNA) or positive controls BubR1 (for reduced mitotic population) and Cdc20 (for increased mitotic population) siRNA. Following a 48‐h incubation, plates were exposed to 10Gy IR and incubated for a further 16 h.

Cells were then washed in PBS using an ELx405 Select Deep Well Washer and then fixed in 3% PFA for 20 min before final PBS washing and stained with pH3 antibody and 5 μg·mL^−1^ DAPI. Plates were then sealed using a PlateLoc Velocity 11 and imaged on a Molecular Devices ImageXpress Micro high‐content microscope using a Multi Wavelength Cell Scoring application on metaxpress (v5.3) to analyse images. The whole screen was carried out three independent times (biological repeats), with each experimental repeat containing three siRNA replicates per 384‐well plate. Potential hits were those that displayed significantly reduced pH3‐positive cells (z‐score > 2) compared to the control siRNA.

### Western blotting

Cells were lysed using RIPA buffer and protein concentrations determined using a Bradford Assay. Lysates were separated by SDS/PAGE and transferred to nitrocellulose. Blots were blocked in 5% milk prior to overnight incubation with specified antibodies at 1 : 1000 in milk.

### Immunofluorescence

HeLa cells (5 × 10^4^) were seeded directly onto coverslips fixed in methanol or paraformaldehyde, permeabilized in 0.2% Triton‐X100, blocked in 5% BSA, and stained with the indicated antibodies. Alexa‐Fluor 488 and Alexa‐Fluor 594 secondary antibodies (Invitrogen) were used. In the final wash, cells were incubated with DAPI (Life Technologies) and mounted to slides using Immu‐mount (Thermo Fisher Scientific). Images were captured using a Nikon ECLIPSE Ti2 confocal microscope.

### Live cell imaging

48 h post transfection with indicated siRNAs, cells were trypsinized, exposed to ionizing radiation (IR) whilst in suspension and reseeded to 24‐well plates. Once adhered to the plates (3–4 h post IR), the cells were loaded into imaging system, or following chemical administration as indicated. Live cell images were captured using ZEISS Cell discoverer 7 microscope every 5 min for a duration of 20 h. ‘Time in mitosis’ was scored from cell rounding to cytokinesis. Any cells that were in mitosis at the start of the experiment or that remained in mitosis beyond the end of the experiment were excluded from final quantification. Likewise, any cell which died during mitosis was excluded as this was found to skew the mitotic transit time.

### PP2a activity assay

The PP2a immunoprecipitation phosphatase assay kit (Sigma‐Aldrich) was used according to the manufacturer's protocol, and absorbance was measured using a Multiskan™ FC Plate Photometer (Thermo Fisher Scientific).

### Comet assay

The neutral comet assay (R&D systems, Minneapolis, MN, USA) was performed according to the manufacturer's protocol; images were captured using a Nikon Eclipse TE200 fluorescent microscope and analysed using the cometscore software.

### Metaphase spreads and analysis

Following treatment, mitotic cells were shaken off into warm KCl (70 mm) and incubated for 10 min at 37 °C. Cells were then loaded into a cytofunnel and centrifuged at 170 ***g*** in the cytospin. Slides were fixed for 10 min in 4% PFA at room temperature, soaked for 10 min in KCM (120 mm KCl, 20 mm NaCl, 10 mm Tris/HCl pH 8.0, 0.5 mm EDTA and 0.1% Triton X‐100) then blocked for 30 min in 5% BSA (37 °C in a humified chamber). Slides were incubated with primary and secondary antibodies at 1 : 100 in 5% BSA for 30 min (37 °C in humidified chamber) with three PBS washes between each step. Slides were incubated with DAPI (Life Technologies), coverslips were mounted, and chromosomes were visualized using a Nikon ECLIPSE Ti2 confocal microscope. Analysis was performed using the image j (fiji) software.

### Statistical analysis

Statistical tests were performed in graphpad prism as described in the figure legends.

## Conflict of interest

The authors declare no conflict of interest.

## Author contributions

NL and RG planned and performed experiments and analysed data. TW wrote the paper and analysed data. SW, PL and CD performed experiments; SB planned experiments and provided reagents and other essential material; RT planned experiments, performed experiments, analysed data, wrote the paper and secured funding for the studies.

## Supporting information


**Video S1.** Time lapse microscopy of untreated HeLa cell. Cells were photographed every 5 min for 16 h.

**Video S2.** Time lapse microscopy of HeLa cell treated with 5Gy IR. Cells were photographed every 5 min for 16 h after exposure to IR.

**Video S3.** Nocodazole‐induced mitotic arrest. HeLa cells were treated with 200 ng·mL^−1^ Nocodazole and were photographed every 5 min for 16 h after exposure.

**Video S4.** Nocodazole‐induced death in mitosis. HeLa cells were treated with 200 ng·mL^−1^ Nocodazole and were photographed every 5 min for 16 h after exposure.

**Video S5.** Nocodazole‐induced mitotic slippage. HeLa cells were treated with 200 ng·mL^−1^ Nocodazole and were photographed every 5 min for 16 h after exposure.

**Video S6.** Time lapse microscopy of siSOD1 untreated HeLa cell. Following 48 h incubation with the indicated siRNA, cells were photographed every 5 min for 16 h.

**Video S7.** Time lapse microscopy of siSOD1 HeLa cell following 5Gy IR. Cells were photographed every 5 min for 16 h after exposure to IR.

## Data Availability

The data that supports the findings of this study are available in Figs 1, 2, 3, 4, 5, 6, 7 and the Supporting Information of this article.

## References

[1] Bartek J , Lukas C & Lukas J (2004) Checking on DNA damage in S phase. Nat Rev Mol Cell Biol 5, 792–804. 15459660 10.1038/nrm1493

[2] Smits VA , Klompmaker R , Arnaud L , Rijksen G , Nigg EA & Medema RH (2000) Polo‐like kinase‐1 is a target of the DNA damage checkpoint. Nat Cell Biol 2, 672–676. 10980711 10.1038/35023629

[3] Mikhailov A , Cole RW & Rieder CL (2002) DNA damage during mitosis in human cells delays the metaphase/anaphase transition via the spindle‐assembly checkpoint. Curr Biol 12, 1797–1806. 12419179 10.1016/s0960-9822(02)01226-5

[4] Rello‐Varona S , Gamez A , Moreno V , Stockert JC , Cristobal J , Pacheco M , Canete M , Juarranz A & Villanueva A (2006) Metaphase arrest and cell death induced by etoposide on HeLa cells. Int J Biochem Cell Biol 38, 2183–2195. 16931106 10.1016/j.biocel.2006.06.013

[5] Thompson R , Shah RB , Liu PH , Gupta YK , Ando K , Aggarwal AK & Sidi S (2015) An inhibitor of PIDDosome formation. Mol Cell 58, 767–779. 25936804 10.1016/j.molcel.2015.03.034 PMC4458193

[6] Blackford AN & Stucki M (2020) How cells respond to DNA breaks in mitosis. Trends Biochem Sci 45, 321–331. 32001093 10.1016/j.tibs.2019.12.010

[7] Kastan MB & Bartek J (2004) Cell‐cycle checkpoints and cancer. Nature 432, 316–323. 15549093 10.1038/nature03097

[8] Giunta S , Belotserkovskaya R & Jackson SP (2010) DNA damage signaling in response to double‐strand breaks during mitosis. J Cell Biol 190, 197–207. 20660628 10.1083/jcb.200911156 PMC2930281

[9] Royou A , Gagou ME , Karess R & Sullivan W (2010) BubR1‐ and polo‐coated DNA tethers facilitate poleward segregation of acentric chromatids. Cell 140, 235–245. 20141837 10.1016/j.cell.2009.12.043 PMC2969851

[10] Leimbacher PA , Jones SE , Shorrocks AK , de Marco Zompit M , Day M , Blaauwendraad J , Bundschuh D , Bonham S , Fischer R , Fink D *et al*. (2019) MDC1 interacts with TOPBP1 to maintain chromosomal stability during mitosis. Mol Cell 74, 571–583.e8. 30898438 10.1016/j.molcel.2019.02.014 PMC6509287

[11] Gomez Godinez V , Kabbara S , Sherman A , Wu T , Cohen S , Kong X , Maravillas‐Montero JL , Shi Z , Preece D , Yokomori K *et al*. (2020) DNA damage induced during mitosis undergoes DNA repair synthesis. PLoS One 15, e0227849. 32343690 10.1371/journal.pone.0227849 PMC7188217

[12] Gelot C , Kovacs MT , Miron S , Mylne E , Haan A , Boeffard‐Dosierre L , Ghouil R , Popova T , Dingli F , Loew D *et al*. (2023) Poltheta is phosphorylated by PLK1 to repair double‐strand breaks in mitosis. Nature 621, 415–422. 37674080 10.1038/s41586-023-06506-6 PMC10499603

[13] Wassing IE , Graham E , Saayman X , Rampazzo L , Ralf C , Bassett A & Esashi F (2021) The RAD51 recombinase protects mitotic chromatin in human cells. Nat Commun 12, 5380. 34508092 10.1038/s41467-021-25643-y PMC8433380

[14] McCord JM & Fridovich I (1969) Superoxide dismutase. An enzymic function for erythrocuprein (hemocuprein). J Biol Chem 244, 6049–6055. 5389100

[15] Barbosa LF , Cerqueira FM , Macedo AF , Garcia CC , Angeli JP , Schumacher RI , Sogayar MC , Augusto O , Carri MT , Di Mascio P *et al*. (2010) Increased SOD1 association with chromatin, DNA damage, p53 activation, and apoptosis in a cellular model of SOD1‐linked ALS. Biochim Biophys Acta 1802, 462–471. 20097285 10.1016/j.bbadis.2010.01.011

[16] Wang XD , Zhu MW , Shan D , Wang SY , Yin X , Yang YQ , Wang TH , Zhang CT , Wang Y , Liang WW *et al*. (2019) Spy1, a unique cell cycle regulator, alters viability in ALS motor neurons and cell lines in response to mutant SOD1‐induced DNA damage. DNA Repair (Amst) 74, 51–62. 30630676 10.1016/j.dnarep.2018.12.005

[17] Hubner NC , Wang LH , Kaulich M , Descombes P , Poser I & Nigg EA (2010) Re‐examination of siRNA specificity questions role of PICH and Tao1 in the spindle checkpoint and identifies Mad2 as a sensitive target for small RNAs. Chromosoma 119, 149–165. 19904549 10.1007/s00412-009-0244-2 PMC2846388

[18] Varadkar P , Abbasi F , Takeda K , Dyson JJ & McCright B (2017) PP2A‐B56gamma is required for an efficient spindle assembly checkpoint. Cell Cycle 16, 1210–1219. 28562161 10.1080/15384101.2017.1325042 PMC5499913

[19] Schmitz MH , Held M , Janssens V , Hutchins JR , Hudecz O , Ivanova E , Goris J , Trinkle‐Mulcahy L , Lamond AI , Poser I *et al*. (2010) Live‐cell imaging RNAi screen identifies PP2A‐B55alpha and importin‐beta1 as key mitotic exit regulators in human cells. Nat Cell Biol 12, 886–893. 20711181 10.1038/ncb2092 PMC3839080

[20] Rao RK & Clayton LW (2002) Regulation of protein phosphatase 2A by hydrogen peroxide and glutathionylation. Biochem Biophys Res Commun 293, 610–616. 12054646 10.1016/S0006-291X(02)00268-1

[21] Juarez JC , Manuia M , Burnett ME , Betancourt O , Boivin B , Shaw DE , Tonks NK , Mazar AP & Donate F (2008) Superoxide dismutase 1 (SOD1) is essential for H_2_O_2_‐mediated oxidation and inactivation of phosphatases in growth factor signaling. Proc Natl Acad Sci U S A 105, 7147–7152. 18480265 10.1073/pnas.0709451105 PMC2438219

[22] Winterbourn CC , Peskin AV & Parsons‐Mair HN (2002) Thiol oxidase activity of copper, zinc superoxide dismutase. J Biol Chem 277, 1906–1911. 11698397 10.1074/jbc.M107256200

[23] Montllor‐Albalate C , Kim H , Thompson AE , Jonke AP , Torres MP & Reddi AR (2022) Sod1 integrates oxygen availability to redox regulate NADPH production and the thiol redoxome. Proc Natl Acad Sci U S A 119, e2023328119. 34969852 10.1073/pnas.2023328119 PMC8740578

[24] Kruse T , Zhang G , Larsen MS , Lischetti T , Streicher W , Kragh Nielsen T , Bjorn SP & Nilsson J (2013) Direct binding between BubR1 and B56‐PP2A phosphatase complexes regulate mitotic progression. J Cell Sci 126, 1086–1092. 23345399 10.1242/jcs.122481

[25] Wang J , Wang Z , Yu T , Yang H , Virshup DM , Kops GJ , Lee SH , Zhou W , Li X , Xu W *et al*. (2016) Crystal structure of a PP2A B56‐BubR1 complex and its implications for PP2A substrate recruitment and localization. Protein Cell 7, 516–526. 27350047 10.1007/s13238-016-0283-4 PMC4930772

[26] Huang H , Hittle J , Zappacosta F , Annan RS , Hershko A & Yen TJ (2008) Phosphorylation sites in BubR1 that regulate kinetochore attachment, tension, and mitotic exit. J Cell Biol 183, 667–680. 19015317 10.1083/jcb.200805163 PMC2582891

[27] Cordeiro MH , Smith RJ & Saurin AT (2020) Kinetochore phosphatases suppress autonomous polo‐like kinase 1 activity to control the mitotic checkpoint. J Cell Biol 219, e202002020. 33125045 10.1083/jcb.202002020 PMC7608062

[28] Carter CD , Kitchen LE , Au WC , Babic CM & Basrai MA (2005) Loss of SOD1 and LYS7 sensitizes *Saccharomyces cerevisiae* to hydroxyurea and DNA damage agents and downregulates MEC1 pathway effectors. Mol Cell Biol 25, 10273–10285. 16287844 10.1128/MCB.25.23.10273-10285.2005 PMC1291217

[29] Tsang CK , Liu Y , Thomas J , Zhang YJ & Zheng XFS (2014) Superoxide dismutase 1 acts as a nuclear transcription factor to regulate oxidative stress resistance. Nat Commun 5, 3446. 24647101 10.1038/ncomms4446 PMC4678626

[30] Bordoni M , Pansarasa O , Dell'Orco M , Crippa V , Gagliardi S , Sproviero D , Bernuzzi S , Diamanti L , Ceroni M , Tedeschi G *et al*. (2019) Nuclear Phospho‐SOD1 protects DNA from oxidative stress damage in amyotrophic lateral sclerosis. J Clin Med 8, 729. 31121901 10.3390/jcm8050729 PMC6572067

[31] Li X , Qiu S , Shi J , Wang S , Wang M , Xu Y , Nie Z , Liu C & Liu C (2019) A new function of copper zinc superoxide dismutase: as a regulatory DNA‐binding protein in gene expression in response to intracellular hydrogen peroxide. Nucleic Acids Res 47, 5074–5085. 31162603 10.1093/nar/gkz256 PMC6547762

[32] Li X , Chen Y , Zhao J , Shi J , Wang M , Qiu S , Hu Y , Xu Y , Cui Y , Liu C *et al*. (2019) The specific inhibition of SOD1 selectively promotes apoptosis of cancer cells via regulation of the ROS signaling network. Oxid Med Cell Longev 2019, 9706792. 30911355 10.1155/2019/9706792 PMC6398008

[33] Nijenhuis W , Vallardi G , Teixeira A , Kops GJ & Saurin AT (2014) Negative feedback at kinetochores underlies a responsive spindle checkpoint signal. Nat Cell Biol 16, 1257–1264. 25402682 10.1038/ncb3065 PMC6485516

[34] Gavet O & Pines J (2010) Progressive activation of CyclinB1‐Cdk1 coordinates entry to mitosis. Dev Cell 18, 533–543. 20412769 10.1016/j.devcel.2010.02.013 PMC3325599

[35] Cervone N , Monica RD , Serpico AF , Vetrei C , Scaraglio M , Visconti R & Grieco D (2018) Evidence that PP2A activity is dispensable for spindle assembly checkpoint‐dependent control of Cdk1. Oncotarget 9, 7312–7321. 29484112 10.18632/oncotarget.23329 PMC5800904

